# Distribution of two-component signal transduction systems BlpRH and ComDE across streptococcal species

**DOI:** 10.3389/fmicb.2022.960994

**Published:** 2022-10-24

**Authors:** Hemendra Pal Singh Dhaked, Indranil Biswas

**Affiliations:** Department of Microbiology, Molecular Genetics and Immunology, University of Kansas Medical Center, Kansas City, KS, United States

**Keywords:** *Streptococcus*, BlpRH, ComDE, two-component signal transduction, *Firmicutes*

## Abstract

Two-component signal transduction (TCS) systems are important regulatory pathways in streptococci. A typical TCS encodes a membrane-anchored sensor kinase (SK) and a cytoplasmic response regulator (RR). Approximately, 20 different types of TCSs are encoded by various streptococci. Among them, two TCSs, in particular BlpRH and ComDE, are required for bacteriocins production and competence development. The SK component of these two TCSs is highly similar and belongs to the protein kinase-10 (HPK-10) subfamily. While these two TCSs are present in streptococci, no systematic studies have been done to differentiate between these two TCSs, and the existence of these pathways in several species of the genus Streptococcus is also unknown. The lack of information about these pathways misguided researchers for decades into believing that the *Streptococcus mutans* BlpRH system is a ComDE system. Here, we have attempted to distinguish between the BlpRH and ComDE systems based on the location of the chromosome, genomic arrangement, and conserved residues. Using the SyntTax and NCBI databases, we investigated the presence of both TCS systems in the genome of several streptococcal species. We noticed that the NCBI database did not have proper annotations for these pathways in several species, and many of them were wrongly annotated, such as CitS or DpiB instead of BlpH. Nevertheless, our critical analyses led us to classify streptococci into two groups: class A (only the BlpRH system) and class B (both the BlpRH and ComDE systems). Most of the streptococcal groups, including bovis, pyogenic, mutans, salivarius, and suis, encode only the BlpRH system. In contrast, only in the mitis and anginosus groups were both the TCS systems present. The focus of this review is to identify and differentiate between the BlpRH and ComDE systems, and discuss these two pathways in various streptococci.

## Introduction

Genus Streptococcus consists of heterogeneous gram-positive bacteria that are mostly facultative anaerobes. Streptococci are ecologically crucial for the commensal microbial flora of animals and humans. Under certain conditions, streptococci cause significant human diseases, such as pneumococcal pneumonia, rheumatic heart diseases, scarlet fever, and glomerulonephritis (Cartwright, 2002; Henriques-Normark and Tuomanen, 2013). Streptococci are also essential for dairy fermentation and as pollution indicators (Tamime and Deeth, 1980; Sinton et al., 1993). Phylogenetically, the genus streptococcus has been classified into seven major groups, mitis, anginosus, bovis, mutans, pyogenic, salivarius, and suis (Shao et al., 2013).

**Figure 1:**
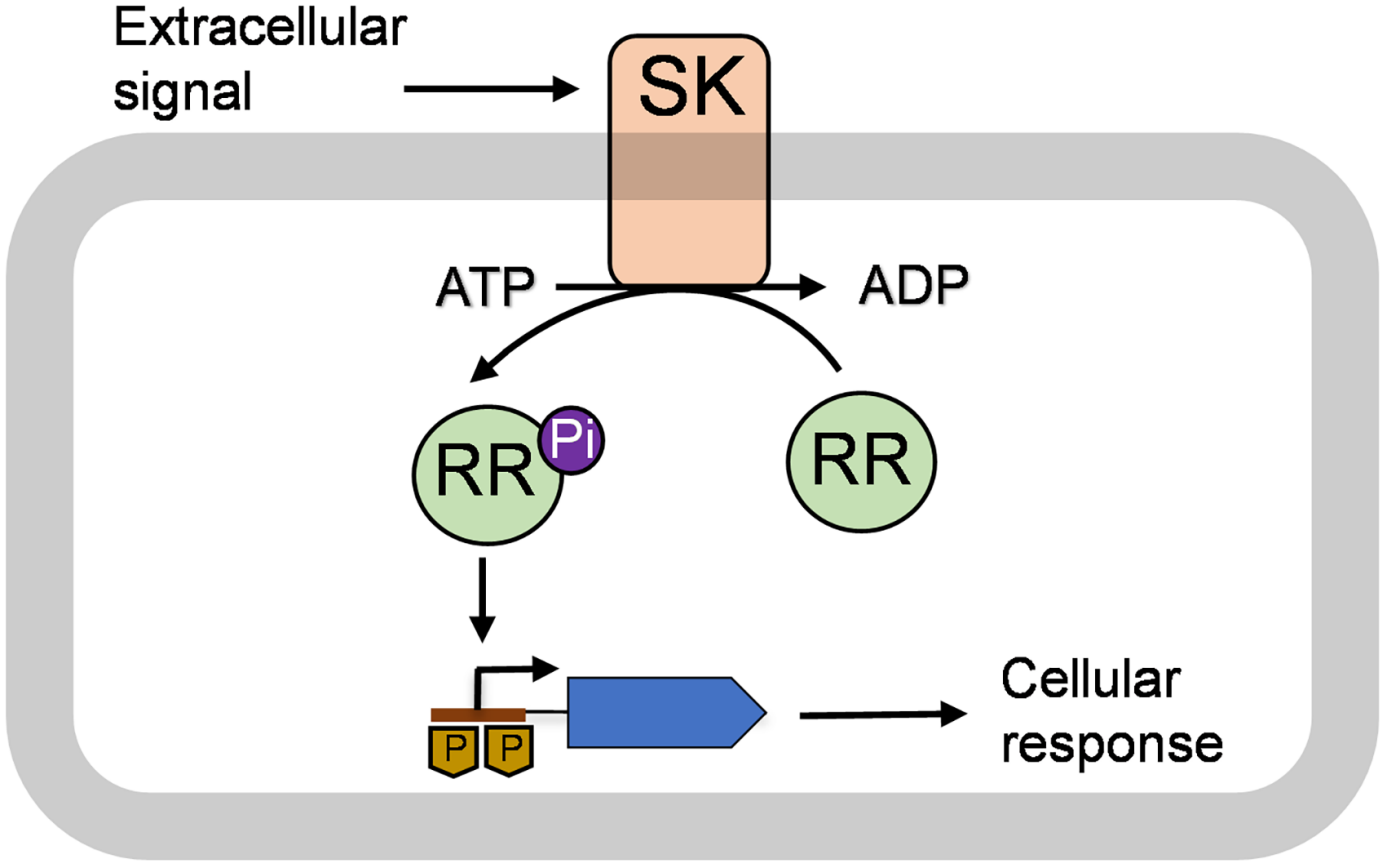
A model of a typical two-component system (TCS) is shown. The TCS consists of a membrane-bound sensor kinase (SK) and a cytoplasmic response regulator (RR; Stock et al., 2000; Galperin, 2004). The SK catalyzes autophosphorylation using adenosine triphosphate (ATP) upon getting a signal from the extracellular environment (Mascher et al., 2006; Casino et al., 2009; Ferris et al., 2012). This signal could be changed in temperature or pH, small molecules, and quorum-sensing peptides. The phosphorylated SK interacts with cognate RR and transfers its phosphoryl group (Pi) to RR (Hoch, 2000; Stock et al., 2000; Mascher et al., 2006; Gao and Stock, 2009; Goulian, 2010; Bhate et al., 2015). The phosphorylated RR binds to cognate promoters and regulates gene production that leads to cellular responses (Gao et al., 2007; Gao and Stock, 2009; Barbieri et al., 2010).

Bacteria including streptococci sense the environment in which they reside and respond to the changes by involving a specialized gene regulatory pathway known as the two-component signal transduction (TCS) system. As shown in Figure 1, a typical TCS system consists of a membrane-anchored sensor kinase (SK) and cytoplasmic RR that generally acts as a transcription factor (Stock et al., 2000; Galperin, 2004). SKs contain two distinct domains: a transmembrane spanning domain and a cytoplasmic domain. The transmembrane domains sense the signal from the external environment and trigger a conformational change in the protein (Mascher et al., 2006; Bhate et al., 2015). This leads to auto phosphorylation of a conserved histidine residue in the cytoplasmic domain using ATP (Mascher et al., 2006; Casino et al., 2009; Ferris et al., 2012). The RR also contains two domains, an N-terminal receiver domain and a C-terminal effector domain, that generally acts as a DNA-binding domain (Walthers et al., 2003; Casino et al., 2009). The phosphorylated SK interacts with the cognate RR and transfers a phospho group to a conserved aspartate group that resides in the receiver domain (Hoch, 2000; Stock et al., 2000; Mascher et al., 2006; Gao and Stock, 2009; Goulian, 2010; Bhate et al., 2015). The phosphorylation of aspartate leads to activation of the RR, which then binds to cognate promoters and modulates expression of genes that are necessary for cellular responses (Gao et al., 2007; Gao and Stock, 2009; Barbieri et al., 2010). The nature of the environmental signals that trigger a TCS system varies drastically. These signals include physical or chemical changes in the environment, such as pH or temperature, and the presence of small molecules or small peptides in the milieu that acts as a quorum-sensing molecule (Stock et al., 2000; Beier and Gross, 2006; Gao and Stock, 2009; Tiwari et al., 2017).

Genomes of streptococci generally encode approximately 20 different TCSs; however, the number varies depending on the species as well as strains. These TCSs are grouped based on the presence of six conserved boxes (H, X, N, D, F, and G boxes) in the SK domains (HPKs; Grebe and Stock, 1999). For example, the pneumococcal genome encodes 13 types of TCS systems that are dispersed all along its chromosomes (Lange et al., 1999; Throup et al., 2000; Gomez-Mejia et al., 2018). Among these TCS systems, TCS 2, 4–6, 8, and 10 belong to the HPK1 subfamily, TCS 1, 3, and 11 belong to the HPK3 subfamily, TCS 7 and 9 belong to the HPK8 subfamily, and TCS 12 and 13 belong to the HPK10 subfamily (Grebe and Stock, 1999; Gomez-Mejia et al., 2018). The streptococcal HPK10 subfamily TCS systems are very important since they are involved in virulence-related attributes and natural competence development. The HPK10 subfamily SKs display several characteristic conserved motifs named as H-box (IRSFRHDYXNILTSLR), N-box (LXDNAIEAA), and G-box (STKGXXRGIGLA). H-box, N-box, and G-box are essential for autophosphorylation and ATP binding (Grebe and Stock, 1999; Dutta and Inouye, 2000; Wolanin et al., 2002). In streptococci, two types of HPK10 subfamilies of TCS systems exist: ComDE (TCS12) and BlpRH (TCS13). These two systems are activated in a cell-density-dependent manner (quorum sensing) involving small peptides as signaling molecules.

Both the BlpRH and the ComDE systems also encode a small peptide of approximately 50 residues long encoded by BlpC or ComC, respectively. These peptides are synthesized as pre-peptides inside the cell. The peptides are then secreted outside by dedicated ABC transporters. During secretion, these peptides are cleaved to remove the N-terminal signal sequences and the mature peptides are accumulated in the milieu (Hale et al., 2005; Shanker and Federle, 2017). In some cases, such as BlpC, the mature peptide is further processed by a dedicated membrane-associated protease called SepM (Hossain and Biswas, 2012; Biswas et al., 2016). When the concentration of the matured peptides reaches a certain density, they trigger the respective TCS pathway (Kreth et al., 2005, 2006; Pinchas et al., 2015).

**Figure 2:**
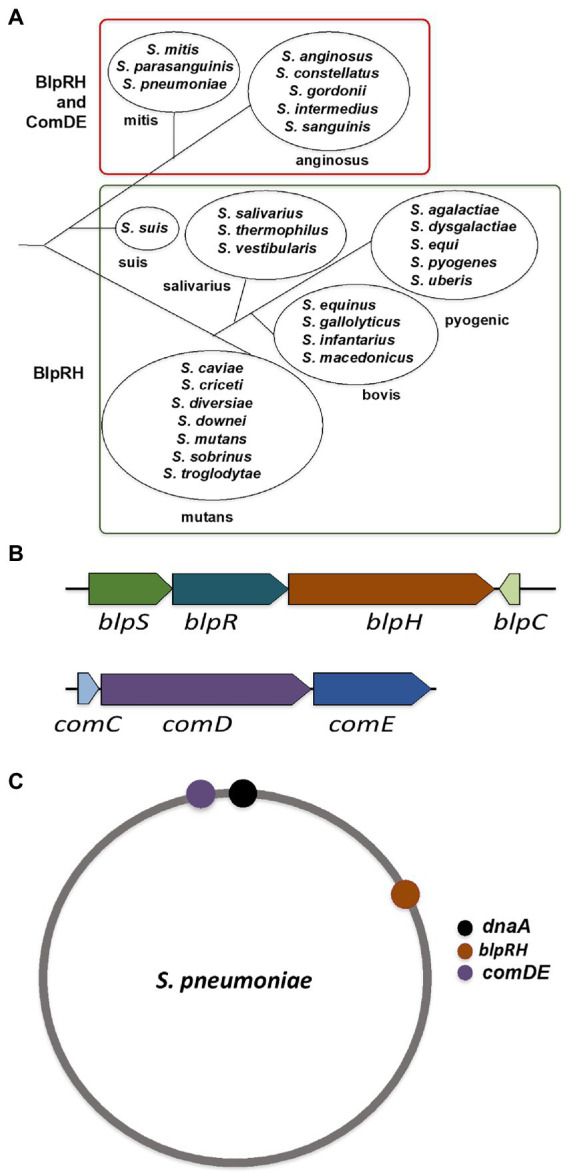
BlpRH and ComDE systems in streptococci. **(A)** Classification of genus Streptococcus based on the presence of BlpRH and ComDE. A phylogenic tree of major species is shown (branch lengths are not to the scale). The groups mitis and anginosus encode both the TCS pathways containing HPK-10 subfamily proteins (BlpH and ComD), classified as a class B group. While the other groups such as suis, mutans, salivarius, bovis, and pyogenic encode only the BlpRH system, classified as a class A group. **(B)** Schematic representation of the operon structure of BlpRH and ComDE systems. Gene annotation is according to the NCBI designation. Note that some BlpRH systems often encode an additional gene, *blpS*, upstream of *blpR*. **(C)** Localization of BlpRH and ComDE systems on the genome of *Streptococcus pneumoniae*. The BlpRH is located on the first quadrant while the ComDE is located on the fourth quadrant, near the origin.

In the literature, a few species of streptococcus are known to possess both the BlpRH and ComDE systems. However, it is not clear whether these pathways are present in specific or all the major phylogenetic groups of the genus streptococcus. One reason is that the differences between the BlpRH and ComDE systems are difficult to distinguish since both are members of the HPK10 subfamily and share similar conservative motifs. Furthermore, in the literature, the BlpRH system is often annotated as the ComDE system (Johnston et al., 2014; Shanker and Federle, 2017). In this review, we have systematically analyzed these two systems and found that the five major streptococcus groups, suis, salivarius, bovis, pyogenic, and mutans, encode only the BlpRH system; while the remaining two major groups, mitis and anginosus, encode both the BlpRH and the ComDE systems (Figure 2A). Furthermore, we were able to differentiate between the two systems based on their gene arrangement, location in the genome, and the conserved amino acid residues present in the BlpH and ComD SKs. In this review, we are emphasizing our key findings based on our *in-silico* analysis.

### Grouping of streptococci based on the presence of the BlpRH and ComDE systems

**Figure 3:**
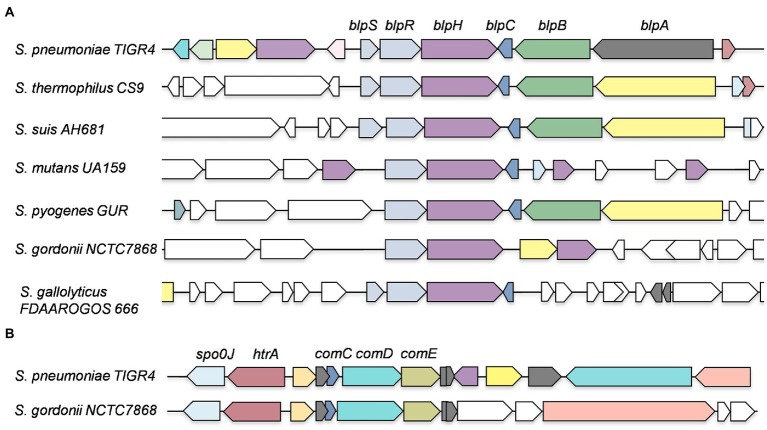
Genomic organization of BlpRH and ComDE pathways in various streptococcal genomes. **(A)** A streptococcal species from each group was selected and SyntTax was performed by using BlpH_SPN_ sequence as an input file. Note that some streptococcal strains encode an additional gene, *blpS*, which has been shown to be necessary for expression. **(B)** A SyntTax profile of ComDE pathways was performed using ComD_SPN_ sequence as an input file.

We used the NCBI nucleotide database and SyntTax to examine the genomes of nearly 100 streptococci (Oberto, 2013). The SyntTax profile of each species was analyzed using BlpR of *S. mutans* (BlpR_SMU_) as the input file. Based on the SyntTax score and the presence of *blpRH* along with *blpC* and *blpS* (which is only present in certain streptococci), we selected at least three genomes in each category when available. The SyntTax profiles provided valuable information regarding surrounding genes and their arrangement in the genome. Several *Streptococcus pneumoniae* BlpH (BlpH_SPN_) corresponding proteins were not named, because they were not identified during annotations and some of the corresponding proteins were named differently than BlpH. We also realized that some of these proteins might not belong to the HPK10 subfamily and some of them might be ComD instead of BlpH. To eliminate these possibilities, we first verified the HPK10 conserved H-box, N-box, and G-box motifs in their amino acid sequences. Afterward, we analyzed the genomic arrangements to differentiate between the BlpRH and ComDE systems. Intriguingly, we found that the gene organization of the *blpRH* and *comDE* genes is very different across the species. In the *blpRH* system, the HK encoding *blpH* gene is encoded downstream of the RR encoding *blpR* gene. The signal peptide encoding the *blpC* gene is present downstream of the *blpH* gene but in the opposite orientation (Figure 2B). In some streptococci, such as *S. gallolyticus* and *S. pneumoniae*, a fourth gene, *blpS,* is also present just upstream of the *blpR* gene. The function of BlpS is not well studied but it appeared to act as a repressor in *S. gallolyticus* (Proutiere et al., 2021). On the other hand, in the *comDE* system, the RR encoding *comE* gene is present downstream of the HK encoding *comD*. The signaling peptide encoding the *comC* gene is present just upstream of the *comD* and transcribes in the same direction as *comD*. Apart from the operon organization, the localization of these two TCS systems on the genome is also different. In *S. pneumoniae*, BlpRH is located in the first quadrant of the genome (Figure 2C). The processing and export encoding ABC transporter genes such as *blpA* and *blpB* are encoded by a nearby region of *blpH* (Figure 3A). The *comDE* locus is located near the origin of replication. A serine protease encoding gene *htrA* and the chromosome partitioning protein encoding gene *spoOJ* are located just upstream of the *comDE* operon (Figures 2C, 3B). Although we considered these two parameters to identify and distinguish between the BlpRH and ComDE pathways, the primary method to differentiate between BlpRH and ComDE systems is genomic arrangement since BlpRH is located around the genome depending on the species (discussed below).

We found approximately 50 streptococcal species encoded only the BlpRH system, whereas nearly 40 other streptococcal species encoded both the BlpRH and ComDE systems (Figure 2A; Supplementary Table S1). As mentioned above, streptococci are phylogenetically classified into seven major groups. Based on the presence or absence of the BlpRH and ComDE systems, we grouped the species into two major classes: A and B (Figure 2A). The class A, which contains only the BlpRH system, contains members of the phylogenetic groups such as suis, salivarius, bovis, pyogenic, and mutans. The class B contains both the BlpRH and ComDE systems and only the anginosus and mitis groups belong to this category. We also noticed that all the streptococci for which genome information is available belong to either of these two categories except for *S. oralis* (belong to mitis group) and *S. sanguinis* (belong to anginosus group). We found that only some of the *S. oralis* and *S. sanguinis* strains, but not all, encode both the BlpRH and ComDE systems (see below).

To delve into the details of the occurrence of the BlpRH systems, we selected the *S. pneumoniae* genome since it is a well-studied species belonging to the mitis group, a relatively diverse group. We performed SyntTax analysis on 150 strains of *S. pneumoniae* using BlpR_SPN_ as an input file. We found that 143 strains had a > 90% SyntTax score and were considered to be in the high score category, five strains had a 50–90% SyntTax score and were considered as a medium score category, and only four strains had an almost 30% SyntTax score, which is very low score category. All the strains from the high score category had all the components of the BlpRH systems. Two strains (NT_110_58 and XDR SMC1710-32) from the medium score category also had the components of the BlpRH pathway. One strain (2245STDY6106637) with a medium score appeared to encode an incomplete BlpR protein (1–125 aa) from the BlpRH pathway; however, this strain encoded BlpS, BlpH, and BlpC. Probably, this strain might encode a functional BlpRH system. The other strain in the medium category (D122) had an undefined BlpR sequence and a fragmented BlpH sequence (due to stop codon) indicating a non-functional BlpRH system. Another strain, SCAID PHRX1-2019, belonging to the medium score class also had a non-functional BlpRH system as its components BlpS, BlpR, BlpH, and BlpC had numerous frameshift mutations. When we compared its *blpH* with *blpH* of TIGR4, we found that though their nucleotide sequences showed 98% identity, the SCAID PHRX1-2019 strain did not have 29 nucleotides positioned from 574th to 602nd in the *blpH* of TIGR4. The strains with the lowest scores (475, 521, 525, and 563) did not show any BlpR_SPN_ homologous proteins. Further, we used the NCBI database to check the presence of the BlpRH system in the genome of these strains. These strains did not encode BlpH and BlpR, but they encoded a putative BlpC. BlpC was present at similar positions in their genome as in the TIGR4 strain, and the BlpC sequence of these four strains was 100% identical to the BlpC of the TIGR4 strain. The location of the BlpRH system on the chromosome in all the strains belonging to the high score and medium score categories was similar.

**Figure 4:**
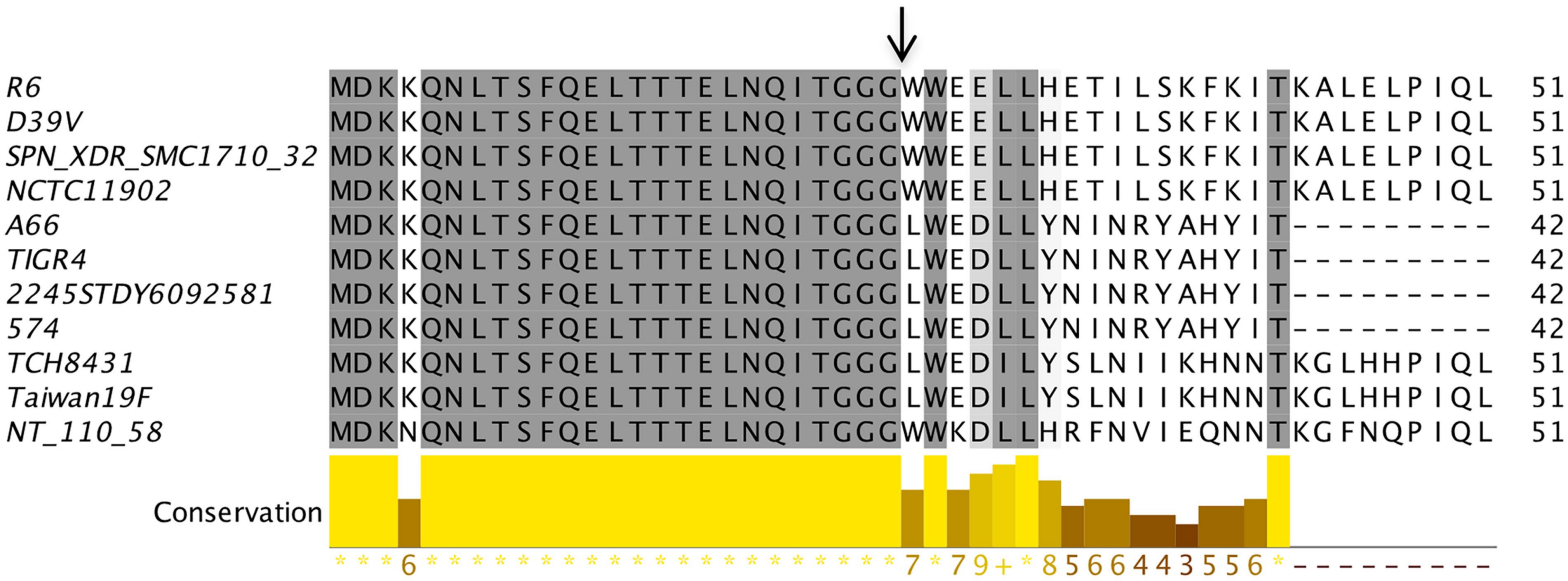
BlpC polymorphism in *Streptococcus pneumoniae*. The multiple sequence alignment of BlpC homologs from different *S. pneumoniae* strains was performed using Muscle with Jalview software. The residues that showed ≥60% conservation were highlighted by grey color. Graph bar represents conservation in each column. Arrow indicates the processing site to generate the mature peptide.

During our analysis, we observed that 28 strains did not have any annotation for the components of the BlpRH system, and 29 strains including NCTC11902 and NT 110 58 were annotated as CitS and one strain, ATCC 49619, was annotated DpiB instead of BlpH. We selected 10 strains from the high score category (including those that showed incorrect annotations) and two strains from the medium score category to verify whether these strains encode identical BlpR or BlpH proteins. The BlpRH system of selected strains was retrieved from the NCBI nucleotide database and translated into the BlpR or BlpH protein sequences. The identities of BlpH and BlpR between the *S. pneumoniae* TIGR4 strains from the other selected strains were determined. The BlpH sequences of strains 574, XDR SMC1710 32, 2245STDY6092581, and A66 showed 95–100% identity with the *S. pneumoniae* TIGR4 strain. The BlpH sequences of strains NCTC11902, NT 110 58, Taiwan19F, and TCH8431 showed 90–95% identity, while the strains ATCC 49619, R6, and D39V showed 85–90% identity with *S. pneumoniae* TIGR4. Surprisingly, the BlpR of these strains were 95–100% identical to the BlpR of the TIGR4 strain. Similarly, the percentage identities between the TIGR4 BlpC and BlpC from other strains were calculated (Figure 4). We found that BlpC peptides of 574, 2245STDY6092581, and A66 strains showed 100% identity with the BlpC of the TIGR4 strain. The BlpC peptides of Taiwan19F and TCH8431 strains showed 67% identity, while other strains NCTC11902, R6, D39V, XDR SMC1710 32, and NT 110 58 showed 59–61% identity with the TIGR4 strain. It seems that the ATCC 49619 strain does not encode any BlpC; therefore, it is not clear whether the BlpRH system is functional or not in this strain. Apart from the sequence similarity in the BlpRH systems of NCTC11902 strain, NT 110 58 strain, and TIGR4 strain, the operon organization of nearby genes and location of this system were similar to the BlpRH system of TIGR4. These analyses led us to believe that annotated CitS is a BlpH homolog, and its phenotype could be similar to the BlpH of the TIGR 4 strain.

*Streptococcus pneumoniae* strains such as 475, 521, 525, and 563 did not encode the components of the BlpRH system, although the ComDE system was present in these strains. The physiological relevance of the lack of the BlpRH system in these strains needs to be experimentally determined. Overall, it appears that the BlpRH system is well conserved across the majority of *S. pneumoniae* strains.

### Evolutionary relationship of BlpH among streptococci

To determine the evolutionary relationship across various streptococci, we constructed a phylogenetic tree of 29 species containing 73 strains. We used Jalview to establish similarities among BlpH homologs of different species within a group and between the groups of streptococci (Waterhouse et al., 2009; Supplementary Figure S1).

The bovis group species, which includes gallolyticus, macedonicus, equinus, and infantarius, formed one cluster together in the phylogenetic tree suggesting the BlpH homologs are very similar. The mutans group species, which includes diversiae, caviae, criceti, sobrinus, and downei, also formed one cluster together in the phylogenetic tree. Interestingly, the bovis group cluster and the mutans group cluster shared a similar ancestor node suggesting that BlpH homologs of these two groups are more similar to each other than the other groups.

We also found that the anginosus group species that include anginosus, intermedius, and constellatus formed a single cluster together in the phylogenetic tree. BlpH homologs of these anginosus species were also similar to some of the pyogenic group species such as agalactiae and equi since both the species share a similar ancestor node in the phylogenetic tree. Similarly, the sanguinis species from the anginosus group encode BlpH homologs that are similar to the pyogenic species pyogenes and dysgalactiae, as well as some suis species strains such as D12 and SRD478. In contrast, gordonii species from the anginosus group and uberis species from the pyogenic group displayed separate ancestral nodes suggesting that BlpH homologs are dissimilar compared to the other groups.

We also noticed that the ancestor node of parasanguinis species from the mitis group and vestibularis species from the salivarius group were the same, suggesting high similarities among the BlpH homologs. Surprisingly, the BlpH homologs of the parasanguinis species were different than the other mitis group species such as mitis, pneumonia, and oralis. It is noteworthy that although oralis species generally encode only the ComDE system, we found at least three strains (ORALIS-351, FDAARGOS_1021, and FDAARGOS_885) that encoded both the BlpRH and ComDE systems.

Taken together, it appears that BlpH homologs of parasanguinis species from the mitis group are more similar to vestibularis from the salivarius group than the other groups.

When we analyzed the BlpH homologs from the salivarius group (salivarius, thermophilus, and vestibularis species), we found that these homologs generated a different ancestral node suggesting a wide variation in their BlpH homologs. Among these, the salivarius species had the most distinct ancestor node. We found that thermophilus strains CNRZ1066 and B59671 encoded identical BlpH protein. The BlpH homologs of the thermophilus strain CS9 and the acidominimus strain NCTC112957 were identical; these BlpH homologs were overall ~69% similar to the BlpH homologs from the mitis group species mitis and pneumoniae. The strains belonging to vestibularis species encoded identical BlpH protein sequence and it is ~78% similar to the BlpH homologs of the parasanguinis species from the mitis group.

### Genetic variation in BlpRH systems across streptococcal species

**Figure 5:**
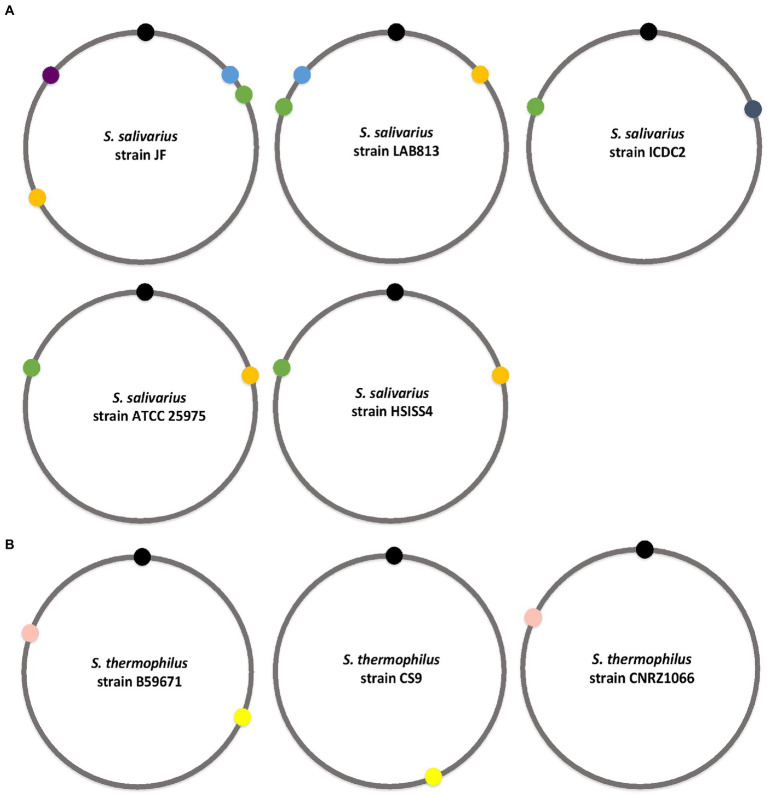
Genomic localization of BlpRH systems in salivarious group. **(A)** Localization of BlpRH systems on the genomes of various *S. salivarius* strains containing two or more BlpRH systems. **(B)** Localization of BlpRH systems in *S. thermophilus* strains. Similar color indicates similar systems. Black circle indicates the position of DnaA (origin).

When we analyzed the NCBI nucleotide data for the presence of the BlpRH system in various streptococcal strains, we found that several strains encode multiple BlpRH systems. The presence of multiple BlpRH systems was more prevalent among the salivarius group than any other groups (Figure 5A). For example, among the *S. salivarius* isolates, JF strain encodes four BlpH homologs (BlpH: AWB63_00870, AWB63_02650, AWB63_07620, and AWB63_07825), LAB813 strain encodes three BlpH homologs (BlpH: FHI56_09880, FHI56_04450, and FHI56_03765), ICDC2 and ATCC 25975 strains encode two BlpH (BSR19_02790, BSR19_08100; and V471_00355, and V471_05055, respectively). Next, we checked for the presence of four BlpH homologs of strain JF in 15 salivarius strains using SyntTax. The first BlpH homolog (AWB63_07825) was present in 5 strains (JIM8777, NCTC8618, 57.I, LAB813, and CCHSS3), the second BlpH homolog (AWB63_07620) was present in all 15 strains, the third BlpH homolog (AWB63_02650) was conserved in 13 strains, and the fourth BlpH homolog (AWB63_00870) was present in only one strain (NCTC8618). Further, we mapped where the identical BlpH homologs were located on the genome of these 15 strains. We found the first BlpH homolog of JF strain, which is present in the first quadrant of the genome, is identical to strain LAB813 BlpH (FHI56_03765), but this BlpH homolog was located in the fourth quadrant of the genome. The second BlpH homolog is also located in the first quadrant of the genome and this BlpH is conserved among all the salivarius strains. The homologs of the second BlpH were identified in strain LAB813 (FHI56_04450), strain ICDC2 (BSR19_08100), strain ATCC 25975 (V471_00355), and strain HSISS4 (HSISS4_01446), but these BlpH homologs were mapped in the fourth quadrant of the genome and not in the first quadrant as in strain JF. The third BlpH homolog was located in the third quadrant of the JF genome and its corresponding BlpH homologs were identified in LAB813 (FHI56_09880), ATCC 25975 (V471_05055), and HSISS4 (HSISS4_00379) strains; but they were located in the second quadrant of the genome in these strains. The fourth BlpH homolog of JF strain was located in the fourth quadrant of genome and its corresponding BlpH homologs were absent in LAB813, ICDC2, ATCC 25975, and HSISS4 strains.

Similar to salivarius, *S. thermophilus* strains also encoded multiple BlpRH systems (Figure 5B). Among 74 publicly available *S. thermophilus* strains, only six strains (ATCC 19258, B59671, DMST H2, NCTC12958, STH CIRM 1048, and STH CIRM 1049) encoded two types of BlpRH systems, located in the second and fourth quadrants in the genome. Among the rest, a single BlpRH system, which is located in the fourth quadrant of the genome of the 66 strains, was present. The remaining two strains (CS9 and KLDS 3.1003) also encode a single BlpRH system, but they are located in the second quadrant of genome. We selected *S. thermophilus* B59671, CS9, and CNRZ1066 strains to determine overall similarity in their BlpRH systems. The strain B59671 encodes two BlpRH systems, CS9 and CNRZ1066, both encode a single BlpRH system. The first BlpH (CG712_09625) of strain B59671 is identical to the BlpH of strain CS9 (DR994_04260) and both BlpH homologs are located in the second quadrant of genome. The second BlpH (CG712_04445) of strain B59671 is identical to BlpH (BlpH: str1687) of CNRZ1066 and they are located in the fourth quadrant. Overall, this analysis showed that some streptococcus strains encode multiple BlpRH systems. Most of these BlpRH systems are partially or completely conserved within the species and the genomic location of these identical BlpRH systems is similar in most of the strains.

We also found that one of the bovis group strains, *S. equinus* FDAARGOS 251, encodes three BlpRH systems (BlpH: A6J79_00355, A6J79_01200, and A6J79_05185) and a mitis group strain, *S. mitis* SVGS_061, encodes two BlpRH systems (BlpH: AXK38_02010, AXK38_05500). Thus, multiple BlpRH systems are often present in streptococci, but the significance of this occurrence is currently unknown.

### Streptococcal strains with an inactivated BlpRH system or missing BlpC signaling peptide

When we translated the nucleotide sequences to identify the BlpRH system, we found that many strains did not encode the predicted and complete BlpH protein, probably due to deletions, insertions or stop codons. For example, *S. uberis* 0140 J displayed a partially predicted BlpH (RS02600) with the missing N-terminus residues (1–132 aa). However, when we checked the intergenic region between the genes RS02595 (predicted to be BlpR) and RS02600, we were able to identify the missing N-terminus region and the translated full-length BlpH sequence of the 0140 J strain showed ~100% identity with the predicted BlpH sequence of another *S. uberis* strain NCTC3858. Similarly, *S. sanguinis* CGMH010 did not encode a complete predicted BlpH protein (FFV08_04910) and the N-terminus region was missing. Therefore, we analyzed the intergenic region between FFV08_04900 (predicted BlpR) and FFV08_04910, and we identified a small protein (109 residues) encoded by FFV08_04905 that shows 49% similarity to respective BlpH TIGR4 thought to be missing from the N-terminus region of predicted BlpH encoded by FFV08_04910. Here, the reason for fragmented BlpH (FFV08_04905 and FFV08_04910) was a stop codon, which was present after the 109th amino acid in small protein FFV08_04905. A similar observation was made for the *S. sanguinis* CGMH058 strain that also lacked the N-terminus region, which we were able to identify by intergenic sequence analysis. We noted that this strain’s BlpH was also present in two parts (FDP16_04785 and FDP16_04790) due to a stop codon but at a different position after the 65th amino acid in FDP16_04785. Interestingly, we found that in the *S. infantarius* CJ18 strain, both the BlpH (Sinf_1717) and the BlpR (Sinf_1716) open reading frames were disrupted completely due to the presence of premature stop codons; however, the BlpC (Sinf_1715) appears to be intact. All the mutations that lead to fragmented or incomplete BlpH were not conserved even within species. At present, we do not know whether these truncated BlpRH sequences are authentic or are due to the sequencing artifact.

Intriguingly, we were unable to detect BlpC peptide in several streptococci isolates belonging to S. agalactiae, *S. caviae, S. diversiae, S. downei, S. equinus, S. gordonii*, *S. mitis, S. oralis, S. salivarius, S. sobrinus, S. suis,* and *S. vestibularis* species. Upon further analysis of the nearby region of BlpRH, we were only able to identify the putative BlpC peptide in *S. downei* and *S. sobrinus*, but not for other species. As mentioned above, in addition we were also unable to find BlpC in some *S. pneumoniae* strains such as ATCC 49619. It is not clear whether these strains truly lack the BlpC peptide or peptides encoded from a different region of the genome might replace the function of BlpC peptide for quorum signaling. Next, we checked the downstream genomic region of BlpH to identify BlpC in other strains. Interestingly, BlpC was found in *S. mitis* SVGS_061, *S. oralis* S MIT Oralis 351, *S. suis* SRD478, *S. equinus* CNU 77–23, and *S. agalactiae* S13 strains. The identified BlpC sequences were similar to BlpC from the other strains within the species.

### Localization of BlpRH systems on streptococcal genome

*Streptococcus pneumoniae* contains a single BlpRH and a single ComDE system, and they are mapped in the first quadrant and fourth quadrant on the genome, respectively (Figure 2C). However, for other species, BlpRH systems are all around the chromosome (Supplementary Table S1). In a majority of the time, the class A group, which contains only the BlpRH (listed in Supplementary Table S1), BlpRH is localized in the fourth quadrant on the genome. In some species, BlpRH is located in the first, second, or the third quadrant on the genome. For example, in bovis group such as *S. equinus*, *S. gallolyticus*, *S. infantarius*, and *S. macedonicus,* BlpRH is present in the fourth quadrant on the genome. On the other hand, in mutans group, BlpRH is mapped at various positions on the genome. In most of the *S. mutans* strains, it is localized in the fourth quadrant. In contrast, the BlpRH of *S. criceti* HS-6, *S. downei* NCTC11391, and *S. sobrinus* strains (6715–7, 6,715–15, and SL1) is mapped in the second quadrant. BlpRH of *S. troglodytae* TKU31 is located in the first quadrant of the genome. In the pyogenic group, the BlpRH of *S. agalactiae, S. equi,* and *S. pyogenes* strains (emm197 and GUR) is present in the fourth quadrant of the genome. However, BlpRH of *S. dysgalactiae* and *S. pyogenes* MGAS8232 is present in the first quadrant on the genome, while BlpRH of *S. uberis* is present on the second quadrant of the genome. In salivarius group, BlpRH systems of *S. salivarius* are mapped in the first, the third (only in JF strain), and the fourth quadrant on the genome. Among the suis group, in *S. suis* strains D12 and SRD478, BlpRH is located in the third quadrant, while, in *S. suis* AH681, it is present in the first quadrant.

For the class B group, which encodes both ComDE and BlpRH systems, the ComDE system is always localized in the fourth quarter of genome, while BlpRH is located at different positions in the genome. For example, in anginosus group, the BlpRH system of *S. anginosus*, *S. constellatus*, and *S. sanguinis* is present in the second quadrant; while for *S. gordonii* and *S. intermedius*, it is present in the first and the third quadrants, respectively. For mitis group, *S. mitis* and *S. pneumoniae*, BlpRH is present in the first quadrant. Among three *S. oralis* strains that encode both ComDE and BlpRH, for strains oralis-351 and FDAARGOS 102, it is present in the first quadrant, while BlpH of FDAARGOS 885 is present in the fourth quadrant. Therefore, the localization of the BlpRH systems on the chromosomes varies between the class A and B groups and within the various phylogenetics groups. We were unable to determine any consensus for the distribution of the BlpRH systems. The genomes of streptococci are known to undergo extensive rearrangements (Nakagawa et al., 2003; Brussow et al., 2004). Thus, it seems that the BlpRH system is localized all around the genome depending on the species.

### Distribution and variation of ComDE system in streptococci

The ComDE system is only present in anginosus and mitis groups, and not in other streptococcal groups. When we analyzed the available genome sequences, we found that unlike the BlpRH system, ComDE is generally mapped in the fourth quadrant of the genomes, near the origin of replication. We also found that, in general, only a single ComDE homolog is present per genome (Supplementary Table S1).

We performed SyntTax on 220 strains from anginosus and mitis groups using ComE_SPN_ as an input. We observed that 170 strains had a > 90% SyntTax score and 47 strains had a 48–90% SyntTax score. The strains (CP2215, gamPNI0373) did not show any ComE_SPN_ homologs. We analyzed the sequences corresponding to ComC, ComD, and ComE of each strain and found that some strains had an incomplete ComDE TCS system.

Deviation from the norm was also observed for some strains of the mitis group. For example, *S. mitis* Nm-65 encodes a ComDE system that mapped in the fourth quadrant on the genome. The ComC and ComE sequences of Nm-65 were 86 and 100% similar to ComC and ComE of TIGR4, respectively. However, ComD of the Nm-65 strain lacked 190 aa at the N-terminus; but the remaining 251 aa of the C-terminus showed 99% similarity with the C-terminus of TIGR4 ComD. When we compared the complete nucleotide sequence (including N-terminus) of *comD* with TIGR4’s *comD*, we found that although *comD* of strain Nm-65 showed 92% nucleotide identity, this *comD* did not have nucleotides located at 33th, 34th, and 208th positions in the *comD* of TIGR4. Some additional nucleotides were also present at the 6th, 205th, and 474th positions in the *comD* of Nm-65. Thus, we speculate the Nm-65 strain encodes an inactive ComDE system and may be non-transformable.

Similarly, we found that the ComDE of *S. pneumoniae* GPSC55 (substr. ST3774) is present in the third quadrant; however, ComC is located in the fourth quadrant. The ComC sequence of this strain is 88% similar to ComC of TIGR4. Surprisingly, we found that ComD is incomplete due to the absence of several nucleotides in the N-terminus region of *comD*. We also found that 16 residues from the C-terminus of ComE were missing due to a stop codon after the 234th aa. The remaining ComE sequences showed 100% similarity to respective ComE sequences from the TIGR4 strain. We found that ComD of *S. pneumoniae* strain 2245STDY5562278 is fragmented due to a stop codon, and translated as two separate proteins, N-domain protein (270 aa) and C-domain protein (275 aa). This might be due to a frameshift error generated during sequencing or could be authentic. The ComC and ComE sequences of this strain were 100% similar to ComC and ComE sequences of TIGR4, respectively. Surprisingly, we found that in some *S. pneumoniae* strains (CP2215 and gamPNI0373), the ComE sequence was missing, while the ComC and ComD sequences were present and located in the fourth quadrant like other ComDE systems. The ComC and ComD sequences of these strains displayed 76–88 and ~ 98% similarity with ComC and ComD sequences of TIGR4, respectively.

Among all the *S. mitis* strains, we found that strains B6, Nm 65, KCOM 1350, NCTC 12261, FDAARGOS 1456, FDAARGOS 684, and SK637 appear to encode two types of ComDE systems. In addition to the canonical ComDE system, which is located on the fourth quadrant on the genome, a second ComDE system is located in the first quadrant. However, this second ComD did not have the characteristic H-box, N-box, and G-box motif residues. In mitis strain B6, the sequences corresponding to the second ComD, ComE, and ComC displayed 53, 56, and 48% similarity with ComD, ComE, and ComC of the canonical ComDE system located in the fourth quadrant.

Another deviation from the canonical ComDE system was found in *S. parasanguinis* FW213 and ATCC 15912 strains. The ComDE system for these two strains is located in the fourth quadrant, similar to TIGR4 and others. However, we found that the ComD protein lacked both the H-box and N-box; and ComC was absent in these two strains. The ComD and ComE sequences of these strain displayed ~53% and ~ 74% similarity with ComD and ComE of TIGR4, respectively.

When we searched the NCBI database for the publicly available 16 *S. oralis* strains (listed in Supplementary Table S1) for the presence of the ComDE and BlpRH systems, we found that only four strains (S.MIT/ORALIS-351, SOD, FDAARGOS_1021, and FDAARGOS_885) encoded both the ComDE and BlpRH systems. The remaining strains (Supplementary Table S1) had only the ComDE system. The similarities among the BlpRH systems in these four strains were very high. We found that the BlpH of strain S.MIT/ORALIS-351 was 91, 89, and 92% similar with strain SOD, FDAARGOS_1021, and strain FDAARGOS_885, respectively. The BlpR of strain S.MIT/ORALIS-351 was ~98% similar to three strains. The S.MIT/ORALIS-351 strain did not have BlpC. The BlpC of strain SOD was 69 and 86%, similar to strain FDAARGOS_1021 and FDAARGOS_885, respectively. These results showed that these four oralis strains had similar BlpRH systems. Furthermore, similarities among the ComDE systems of these 16 strains were also observed. We found that the ComD of strain S.MIT/ORALIS-351 was 88–100% similar with ComD of these 15 strains. The ComE of strain S.MIT/ORALIS-351 was 99–100% similar with ComE of these 15 strains. The ComC of strain S.MIT/ORALIS-351 was 67–100% similar with ComC of these 15 strains. These results showed that these 16 strains had highly similar ComDE systems.

Similar to oralis group, when we performed SyntTax analysis of the ComDE and BlpRH systems on the publicly available genomes of *S. sangunis* strains (CGMH010, CGMH058, FDAARGOS770, NCTC10904, NCTC11085, NCTC11086, NCTC7863, and SK36), we found that all these strains encode a single ComDE system, which was located in the fourth quadrant of the genomes. Surprisingly, we found that BlpRH system was present in only two strains (CGMH010 and CGMH058) and was located in the second quadrant of the genome similar to other anginosus strains.

Although ComDE and BlpRH are the only two TCS systems in streptococci that have the HPK10 SK, we found that some strains of *S. equinus*, which belongs to the phylogenetic group bovis, encode an additional TCS system with more than one SK. For example, the additional TCS systems of NCTC8133, NCTC8140, and MDC1 strains contain three SKs and a single RR within the same operon, while the CNU G6 strain contains two SKs and a single RR. Strain CNU 77–23 contains four SKs and a single RR. A single TCS system with multiple SKs is not unusual and these systems have been found in *Bacillus subtilis* and other organisms (Jiang et al., 2000; Laub and Goulian, 2007; Norsworthy and Visick, 2015).

### Similarities and dissimilarities between BlpH and ComD

**Figure 6:**
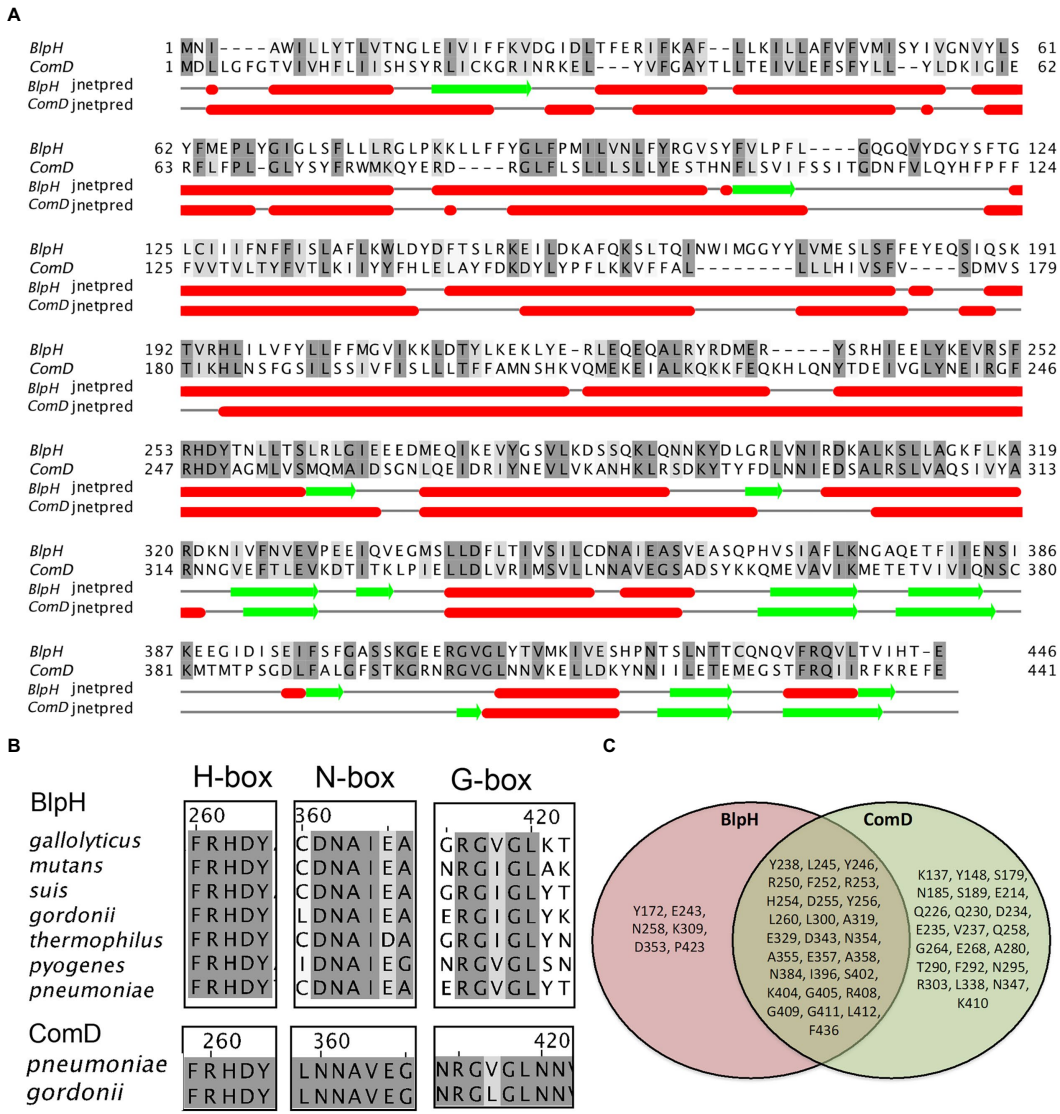
Comparison of conserved motifs and secondary structure of BlpH and ComD sensor kinases. **(A)** The amino acid sequence alignment of BlpH_SPN_ and ComD_SPN_ was performed using Muscle. The residues that showed ≥60% conservation were highlighted with grey color. Secondary structures of BlpH and ComD were predicted separately using Jnetpred in Jalliew. Red cylinders and green arrows represent secondary structures of helices and sheets, respectively. **(B)** Alignment of H-box, N-box, and G-box motifs of BlpH and ComD sensor kinases. The multiple sequence alignment of BlpH homologs from different streptococcal groups was performed as described above. Highly conserved residues in the H-box, N-box, and G-box, which are characteristics of the HPK-10 family, are highlighted in grey. **(C)** A Venn diagram illustrating the common and non-common conserved residues in BlpH and ComD. The multiple sequence alignment of BlpH and ComD homologs from different streptococcal species was performed as described above. The common conserved residues between BlpH_SPN_ and ComD_SPN_ homologs are mentioned in overlapping circle. The residues that are completely conserved in only BlpH or only ComD homologs are mentioned in separate circles.

Both BlpH and ComD belong to the HPK-10 subfamily, and in *S. pneumoniae*, they share 47% sequence similarity (Figure 6A). The conserved motifs H-box, N-box, and G-box characteristics of the HPK-10 family are shown in Figure 6B. We performed multiple sequence alignments (MSA) of 73 strains from 29 streptococcal species (Supplementary Figure S2) and found that there are several conserved residues between BlpH_SPN_ and ComD_SPN_ proteins, which are Y238, L245, Y246, R250, F252, R253, H254, D255, Y256, L260, L300, A319, E329, D343, N354, A355, E357, A358, N384, I396, S402, K404, G405, R408, G409, G411, L412, and F436 (Figure 6C). Despite the motif similarities, both proteins have unique characteristics in their conserved sequences. MSA analysis showed that BlpH_SPN_ contains several residues (Y172, E243, N258, K309, D353, and P423) that are completely conserved in only BlpH homologs from all the streptococcal strains. Similarly, ComD_SPN_ bears residues (K137, Y148, S179, N185, S189, E214, Q226, Q230, D234, E235, V237, Q258, G264, E268, A280, T290, F292, N295, R303, L338, N347, and K410) that are completely conserved only among ComD homologs. The biological significance of these conserved residues is currently unknown.

**Figure 7:**
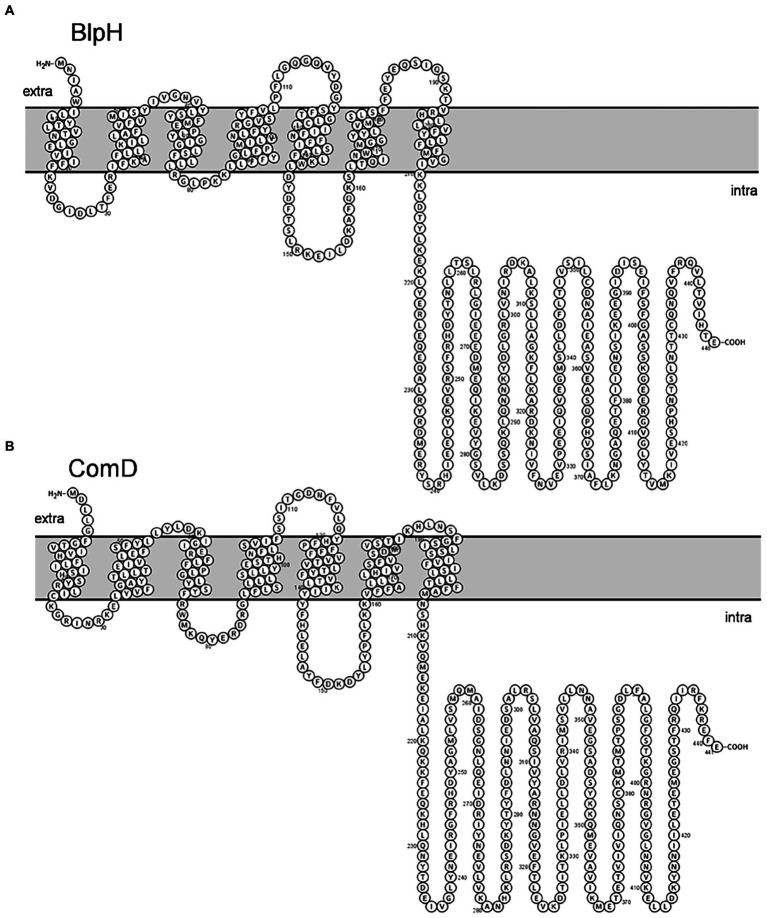
Membrane topology of **(A)** BlpH and **(B)** ComD. Topology was determined using Protter. Note that both BlpH and ComD contain three extracellular loops that are involved in signal recognition.

Further, membrane topology of both ComD and BlpH was determined using Protter. We found that both BlpH and ComD contain three extracellular loops that might be involved in signal recognition (Figure 7).

### BlpC and ComC polymorphism across streptococci

Streptococci have different types of signaling peptides for activating TCS systems. For example, *S. pneumonia*e encodes nearly 21 different types of peptides that are grouped into three categories: double-glycine (GG) peptides, RRNPP peptides, and lanthionine-containing peptides (Aggarwal et al., 2020). These peptides are required for genetic diversity, microbial competition, and environmental adaptions. Among these categories, GG peptides BlpC or ComC activate HPK10 subfamily proteins BlpH and ComD, respectively. Both the peptides are synthesized as pre-peptides with a conserved N-terminal leader sequence containing a conserved GG (or sometimes GA or GS) motif (Havarstein et al., 1995; Aggarwal et al., 2020). BlpC and ComC pre-peptides are cleaved at the GG motif and the cleaved peptide is exported to an external environment by dedicated ATP-binding cassette (ABC) exporters BlpAB and ComAB, respectively (Havarstein et al., 1995; Aggarwal et al., 2020). The N-terminal region of the pre-peptides is more conserved than the C-terminal region. It is thought that the conserved N-terminal domain is required for the processing of the pre-peptides through various exporters. In contrast, the highly variable C-terminal region is needed for the induction of the specific cognate SKs. In many cases, one of the exporters is nonfunctional due to frameshift mutation. In such cases, the peptides are exported through the other exporters. For example, some *S. pneumoniae* strains use the ComAB exporter for BlpC secretion instead of a BlpAB exporter if it is nonfunctional (Kjos et al., 2016; Wholey et al., 2016; Wang et al., 2018). This appears that exporters are redundant for the processing of peptides. The cleaved C-terminal of peptides is the mature products that interact with the cognate SKs to activate the TCS pathway. The role of other GG peptides in activating the BlpRH or ComDE systems is not known.

**Figure 8:**
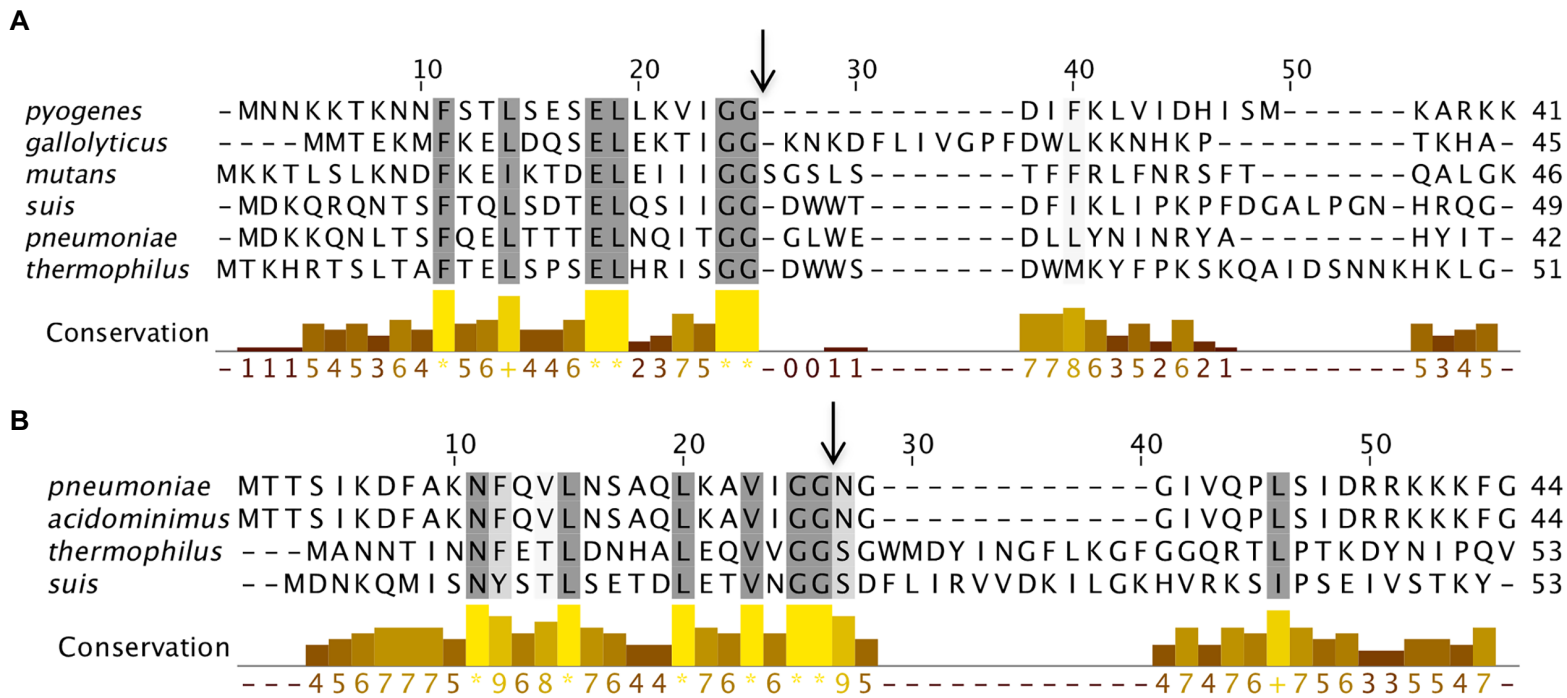
BlpC polymorphism from six streptococcus groups. **(A)** Multiple sequence alignment of BlpC homologs from six different streptococcal groups was performed using Muscle. The residues that showed ≥60% conservation were highlighted by grey color. Graph bar represents conservation in each column and arrow indicates the processing site to generate mature peptide. **(B)** Multiple sequence alignment of a variant of BlpC that is present in some streptococcal species.

When we performed SyntTax analysis of BlpC peptides from various strains, we found that the N-terminus is highly conserved than the C-terminus region across the seven phylogenetic groups. Interestingly, the N-terminus of BlpC has some complete conserved residues F10, L13, E17, L18, G23, and G24 (Figure 8A). Some species such as *S. pneumoniae*, *S. acidominimus*, *S. thermophilus*, and *S. suis* encode two different types of BlpC peptides (Figure 8B). The second category BlpC also contains the GG motif and a different conserved N-terminus region. Furthermore, for several streptococcal strains, we were unable to find a putative BlpC peptide in the nearby region of the BlpRH operon. We speculate that in those strains, the BlpRH pathway is either constitutively active or inactive; other noncognate SKs interact with BlpR for activation. It is also possible that the BlpC is located elsewhere in the genome or other non-BlpC peptides are involved with the signaling.

**Figure 9:**
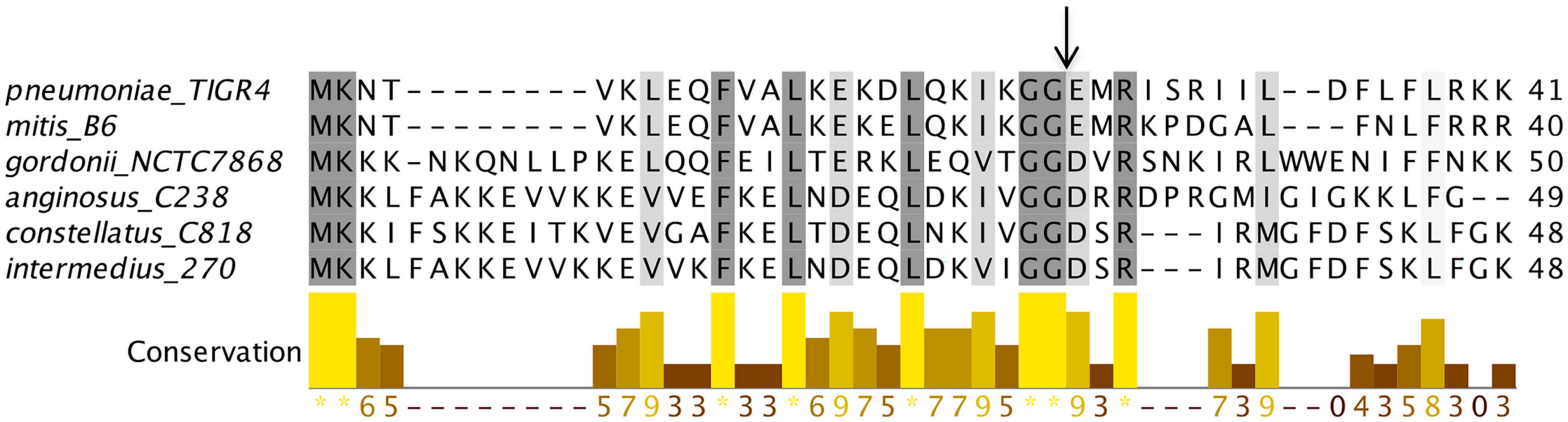
ComC polymorphism among streptococcus species. ComC homologs from representative streptococcal species were used for multiple sequence alignment. Note that for some species, matured ComC sequences show high identity.

Similar to BlpC peptide, ComC peptide displays a high degree of variation across different streptococci as reported before (Whatmore et al., 1999; Salvadori et al., 2019). However, like the BlpC, the N-terminal region is highly conserved with residues such as F10, L13, L18, G23, G24, and R26, and is present in different streptococcal species (Figure 9).

## Materials and methods

### Identification of BlpH/ComD systems

The SyntTax and NCBI nucleotide databases were used to identify BlpRH/ComDE systems in the genomes of streptococci. SyntTax is a web service used to study the conservation of gene order in archaeal and bacterial genomes. This web tool evaluates the orthology of genomic regions in different species/strains and predicts a functional relationship between genes. This web tool allows organism selection based on taxonomy. The minimal normalized genomics BLAST scores indicate the degree of orthology. For example, >90% score shows >90% of orthology between genomic regions of selected strains. The SyntTax^1^ profile of available strains for each streptococcus species (mentioned in Figure 2A) was observed separately using default parameters (Best search in parameters and 10% normalized blast in minimal score were selected) keeping BlpR of *S. mutans* UA159 (BlpR_SMU_) as the input file. The SyntTax produces syntenies (conservation of gene order) for selected genomes. If syntenies did not show BlpR_SMU_ homologous proteins for species, then BlpH_SMU_, BlpR_SPN_, or BlpH_SPN_ were used as the input file. The syntenies were analyzed and at least three strains were selected for each species based on the score and the presence of *blpRH* along with *blpC* and *blpS*. The genomic arrangement of BlpRH was considered to differentiate BlpRH from the ComDE system, as explained above and shown in Figure 2B. Further, the genome of selected strains was investigated to find the location and protein sequences of these systems. The BlpH and BlpR were examined separately with BlpH and BlpR of *S. mutans* UA159 and *S. pneumoniae* TIGR4 by pairwise sequence alignment^2^ to find HPK-10 conservative motifs (H-box, N-box, and G-box). The location of the BlpRH system was noted, considering the *dnaA* first gene (*dnaA* was not present as a first gene in some genomes). These analyses confirmed that identified system is BlpRH or TCS-12, not the other TCS. The ComDE systems were identified using a similar method, except ComE_SPN_ or ComD_SPN_ were used as an input file in the SyntTax web service. The number of strains analyzed for each species are: 9 (*S. equinus*), 9 (*S. gallolyticus*), 2 (*S. infantarius*), 2 (*S. macedonicus*), 1 (*S. caviae*), 1 (*S. criceti*), 1 (*S. diversiae*), 2 (*S. downei*), 39 (*S. mutans*), 6 (*S. sobrinus*), 1 (*S. troglodytae*), 124 (*S. agalactiae*), 63 (*S. dysgalactiae*), 41 (*S. equi*), 261 (*S. pyogenes*), 4 (*S. uberis*), 18 (*S. salivarius*), 74 (*S. thermophilus*), 3 (*S. vestibularis*), 109 (*S. suis*), 13 (*S. anginosus*), 8 (*S. constellatus*), 13 (*S. gordonii*), 8 (*S. intermedius*), 8 (*S. sanguinis*), 10 (*S. mitis*), 150 (*S. pneumoniae*), 21 (*S. oralis*), and 3 (*S. parasanguinis*). Among these species, we found that >78% of strains for each species had BlpRH homologs in mitis group, anginosus group (*S. constellatus* and *S. intermedius*), salivarius group, bovis group, mutans group, and pyogenic group (*S. agalactiae* and *S. equi*). It was also noted that ~50 to ~65% of strains for *S. anginosus*, *S. gordonii*, *S. suis*, *S. dysgalactiae*, and *S. uberis* had BlpRH homologs. For the remaining species, *S. sanguinis*, *S. oralis*, and *S. pyogenes*, only ~20 to ~30% of strains had BlpRH homologs. Interestingly, 100% of strains for the mitis group and anginosus group species had a ComDE system.

## Conclusion

In this study, we analyzed publicly available complete genome sequences to evaluate distribution of two HPK-10 family of TCSs in streptococci. Our analysis revealed that the presence of BlpRH and ComDE varies greatly among streptococci. We found that the ComDE system is specifically present only in two phylogenetic groups of streptococci; whereas, the BlpRH system is present in all the phylogenetic streptococci groups. However, we also found that some streptococcal strains encode multiple ComDE or BlpRH systems that are located all around the genomes. The ComDE system is specifically needed for the development of competence, whereas the BlpRH system modulates production of bacteriocin (Zu et al., 2019). Streptococci that encode both these systems and expression of these two pathways are highly coordinated (Shanker and Federle, 2017; Zu et al., 2019). However, with the strains that encode multiple ComDE systems, it is not known whether all the pathways are necessary for the regulation of competence development. Similarly, the exact roles of encoding multiple BlpRH systems are currently unknown; although one can speculate that each BlpRH system might be involved in regulation of a specific set of bacteriocins. While we identified multiple systems in many streptococcal strains, we are not certain whether all the systems are active or some are pseudogenes. Of note, we found several TCS systems have been inactivated by frame-shift mutations.

Sometimes a particular TCS system can be induced by another TCS system or by an analogous system from a different strain, a phenomenon known as cross-talk (Laub and Goulian, 2007; Podgornaia and Laub, 2013; Agrawal et al., 2016). Cross-talk can happen when a signal is recognized by a non-cognate TCS system or a SK phosphorylates a non-cognate RR. Generally, cross-talk among peptide-mediated induction of a TCS system is uncommon. A recent study in *S. pneumoniae* has analyzed over 4,000 complete and partial genome sequences and found that BlpC is highly polymorphic with nearly 10 different mature BlpC peptides (Miller et al., 2016). Furthermore, the study also found these variant peptides can cross-talk to non-cognate BlpH. However, other studies have shown that the electrical charge of residue 14 of mature BlpC is crucial for specificity (Pinchas et al., 2015; Miller et al., 2018). It appears that other residues in the matured BlpC peptides are also important for binding to BlpH since the various variants with the identical residue 14 differentially activate BlpH.

In *S. thermophilus*, activity of two BlpC variants was studied in LMD-9, LMG18311, and CNRZ1066 strains. The variants only differed in the last C-terminal amino acid. These strains only encode a single BlpRH system. Interestingly, previous study found that both the matured BlpC variants are active (Fontaine et al., 2007). Furthermore, the study also unraveled that the matured BlpC, which is 30 residues long, is further processed upon secretion to generate a 19-residue peptide that is also capable of activating the BlpRH system. Thus, the BlpRH system of *S. thermophilus* is analogous to the *S. mutans* BlpRH system, where the signaling peptide (BlpC) is also further processed from 21 to 18-mer after secretion (Petersen et al., 2006; Hossain and Biswas, 2012). In contrast to the BlpRH system, *S. pneumoniae* ComDE is more restrictive. So far, two allelic variants of CSP have been identified in *S. pneumoniae* and they are unable to cross-talk to one other. On the other hand, *S. mitis,* which also belongs to the same phylogenetic group of *S. pneumoniae,* displays extensive polymorphism in the CSP sequence within the species (Salvadori et al., 2019). Apparently, the cross-talk among various *S. mitis* strains are also not evident (Johnsborg et al., 2008; Salvadori et al., 2019). But in the rare case scenario, it has been demonstrated that the *S. pneumoniae* R6 strain can responds to CSPs produced by two *S. mitis* strains (Johnsborg et al., 2008). However, when a strain encodes two similar CSP peptides, as is the case for some BlpC, it is not known whether both the peptides are able to activate the TCS.

The genomes of streptococci are known to undergo extensive rearrangements and horizontal gene transfer plays a critical role in the evolution of streptococcal genomes (Darmon and Leach, 2014; Richards et al., 2014; Salvadori et al., 2019). Since different streptococci often share the same environmental niche such as the oral cavity (Mundt, 1982; Dhotre et al., 2015; Abranches et al., 2018), it is possible that some strains might have acquired multiple BlpRH or ComDE systems by horizontal gene transfer either by natural transformation or by other means for their benefits. The identification of multiple systems also provides an opportunity to study how streptococcal social networks function in each environmental niche.

## Author contributions

All authors listed have made a substantial, direct, and intellectual contribution to the work and approved it for publication.

## Funding

This work was supported in part by the following grants: DE026955 and DE031455 from the NIDCR/NIH.

## Conflict of interest

The authors declare that the research was conducted in the absence of any commercial or financial relationships that could be construed as a potential conflict of interest.

## Publisher’s note

All claims expressed in this article are solely those of the authors and do not necessarily represent those of their affiliated organizations, or those of the publisher, the editors and the reviewers. Any product that may be evaluated in this article, or claim that may be made by its manufacturer, is not guaranteed or endorsed by the publisher.

## Footnotes

^1^ https://archaea.i2bc.paris-saclay.fr/SyntTax/

^2^ https://www.ebi.ac.uk/Tools/psa/emboss_needle/
