# Supplementary figures and images for: Distribution of two-component signal transduction systems BlpRH and ComDE across streptococcal species
Source: Front Microbiol. 2022 Oct 24;13:960994. doi: 10.3389/fmicb.2022.960994 (PMC9638458; doi:10.3389/fmicb.2022.960994)

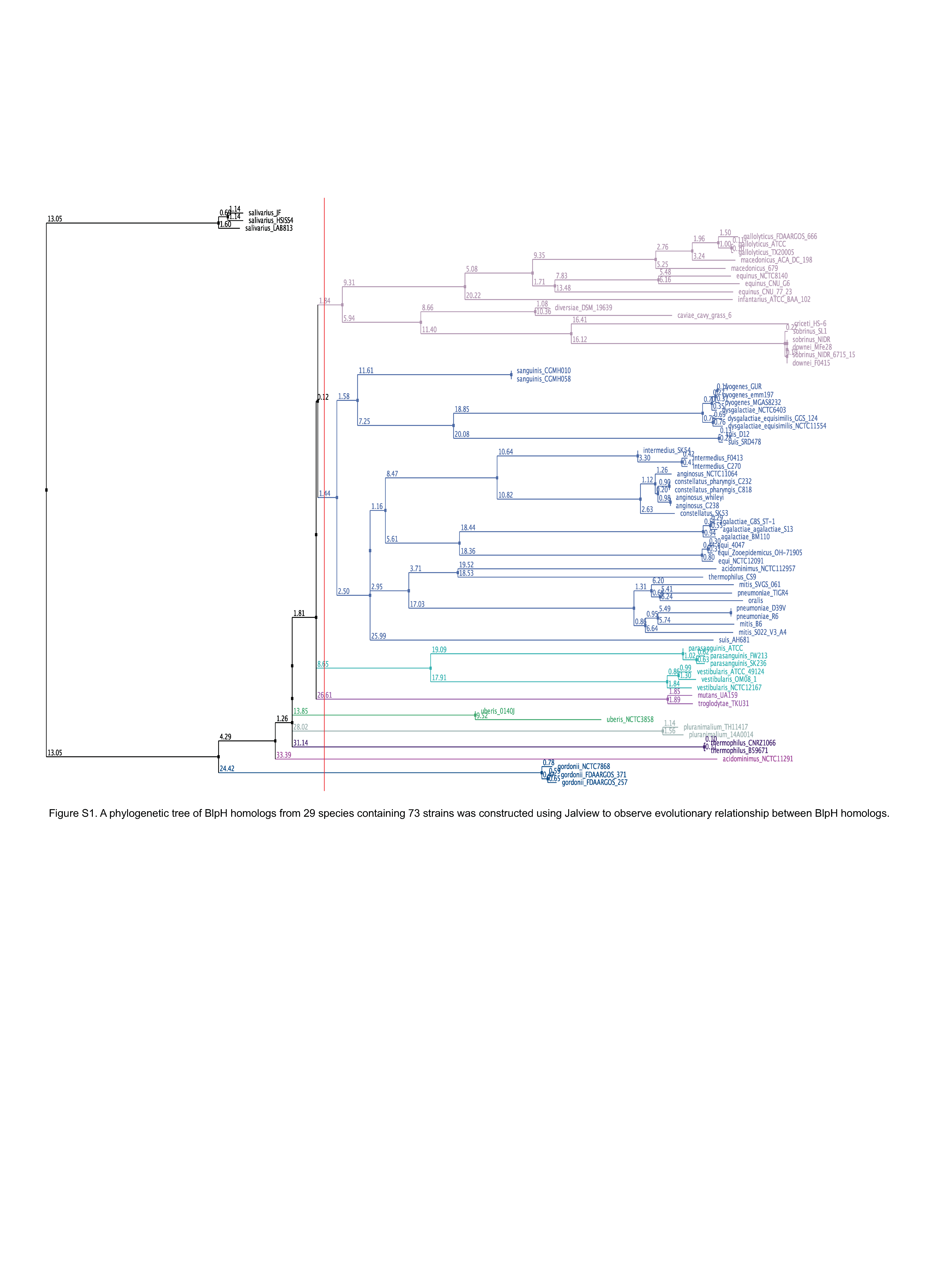

Supplement: Supplementary file 2 [file Image_1.TIFF]

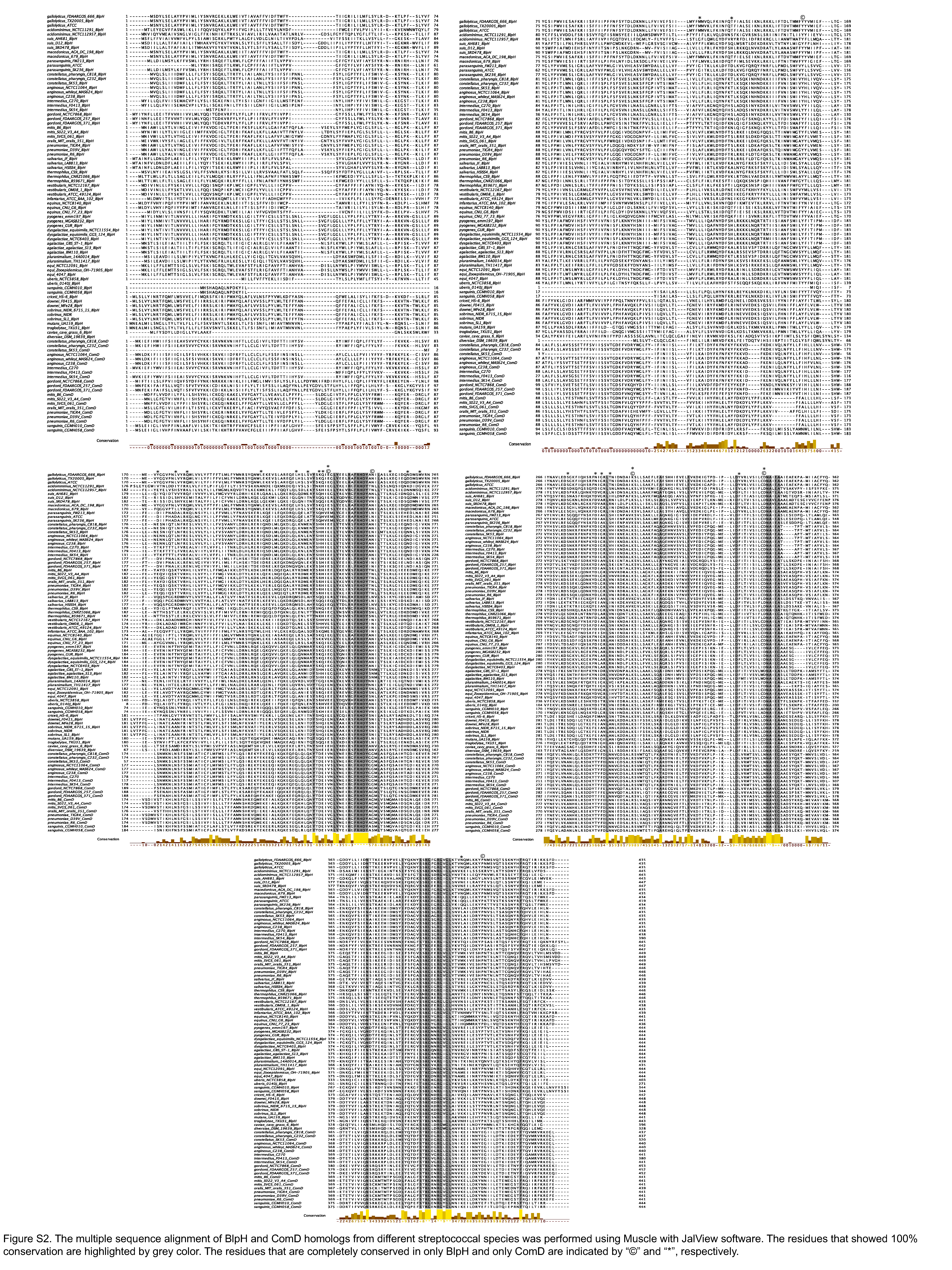

Supplement: Supplementary file 3 [file Image_2.TIFF]
